# Entomopathogenic nematodes as an effective and sustainable alternative to control the fall armyworm in Africa

**DOI:** 10.1093/pnasnexus/pgae122

**Published:** 2024-04-16

**Authors:** Patrick Fallet, Didace Bazagwira, Livio Ruzzante, Geraldine Ingabire, Sacha Levivier, Carlos Bustos-Segura, Joelle Kajuga, Stefan Toepfer, Ted C J Turlings

**Affiliations:** Laboratory of Fundamental and Applied Research in Chemical Ecology, Institute of Biology, University of Neuchâtel, CH-2000 Neuchâtel, Switzerland; CABI-Switzerland, c/o Plant Protection and Soil Conservation Directorate, HU-6800 Hodmezovasarhely, Hungary; Rwanda Agriculture and Animal Resources Development Board, Entomopathogenic Nematodes Production Facility, 5016 Rubona, Rwanda; Laboratory of Fundamental and Applied Research in Chemical Ecology, Institute of Biology, University of Neuchâtel, CH-2000 Neuchâtel, Switzerland; Rwanda Agriculture and Animal Resources Development Board, Entomopathogenic Nematodes Production Facility, 5016 Rubona, Rwanda; Laboratory of Fundamental and Applied Research in Chemical Ecology, Institute of Biology, University of Neuchâtel, CH-2000 Neuchâtel, Switzerland; Laboratory of Fundamental and Applied Research in Chemical Ecology, Institute of Biology, University of Neuchâtel, CH-2000 Neuchâtel, Switzerland; Rwanda Agriculture and Animal Resources Development Board, Entomopathogenic Nematodes Production Facility, 5016 Rubona, Rwanda; CABI-Switzerland, c/o Plant Protection and Soil Conservation Directorate, HU-6800 Hodmezovasarhely, Hungary; MARA-CABI Joint Laboratory for Biosafety, Institute of Plant Protection, Chinese Academy of Agricultural Sciences, 1008641 Beijing, China; Laboratory of Fundamental and Applied Research in Chemical Ecology, Institute of Biology, University of Neuchâtel, CH-2000 Neuchâtel, Switzerland

**Keywords:** biological control, integrated pest management, sustainable agriculture, food security, invasive pest

## Abstract

The recent invasion of the fall armyworm (FAW), a voracious pest, into Africa and Asia has resulted in unprecedented increases in insecticide applications, especially in maize cultivation. The health and environmental hazards posed by these chemicals have prompted a call for alternative control practices. Entomopathogenic nematodes are highly lethal to the FAWs, but their application aboveground has been challenging. In this study, we report on season-long field trials with an innocuous biodegradable gel made from carboxymethyl cellulose containing local nematodes that we specifically developed to target the FAW. In several Rwandan maize fields with distinct climatic conditions and natural infestation rates, we compared armyworm presence and damage in control plots and plots that were treated with either our nematode gel formulation, a commercial liquid nematode formulation, or the commonly used contact insecticide cypermethrin. The treatments were applied to the whorl of each plant, which was repeated three to four times, at 2-week intervals, starting when the plants were still seedlings. Although all three treatments reduced leaf damage, only the gel formulation decreased caterpillar infestation by about 50% and yielded an additional ton of maize per hectare compared with untreated plots. Importantly, we believe that the use of nematodes can be cost-effective, since we used nematode doses across the whole season that were at least 3-fold lower than their normal application against belowground pests. The overall results imply that precisely formulated and easy-to-apply nematodes can be a highly effective, affordable, and sustainable alternative to insecticides for FAW control.

**Significance Statement**

Entomopathogenic nematodes are commonly used as biological control agents against soil pests, but are seldomly used to control foliar insects due to the nematodes’ high sensitivity to aboveground abiotic stressors such as desiccation and ultraviolet radiation. We developed an innocuous gel formulation specifically designed to protect and apply nematodes to maize whorls to control the fall armyworm, a particularly voracious invasive pest of maize. We demonstrated, under realistic farming conditions, that applying the gel formulation every two weeks can successfully control armyworms and increase yields when compared with untreated control plants. The formulation provides new opportunities to contribute to mitigating the impacts of pest control practices on the environment and public health, commonly associated with conventional synthetic insecticides.

## Introduction

The fall armyworm (FAW; *Spodoptera frugiperda*, Smith; Lepidoptera: Noctuidae) is a major pest of maize (corn, *Zea mays*). Originating from the Americas, it has recently spread into Africa and Asia (1–3), causing severe plant damage and tremendous yield losses (4–6). Its invasion threatens the livelihoods of millions of farmers (4, 7, 8) and has led to an excessive reliance on chemical insecticides (6, 9). The environmental and health concerns associated with pesticides have prompted an urgent need for devising safer and more sustainable control measures (4, 6). Several biocontrol agents, including entomopathogenic nematodes (EPNs), have emerged as alternatives to chemical insecticides (6, 10, 11).

EPNs are minute soil-dwelling roundworms found worldwide, except in Antarctica (12). They exclusively parasitize insects, which they locate with the help of chemical cues (13) and enter via natural orifices, or in some cases through the cuticle (14, 15). EPNs carry symbiotic bacteria in their guts that are released inside their host (14, 15). These bacteria, as well as the EPN, exude toxins that rapidly kill their insect host (14, 15). EPNs can kill a large variety of insects and can be easily mass-produced (16), also in Africa (17). They are safe for humans and the environment (18) and have been successfully used as biocontrol agents against belowground insect pests (19). Although EPNs are sensitive to ultraviolet (UV) radiation and desiccation (14, 20, 21), the protective location of the feeding FAW caterpillars, mostly deep within the maize whorl (1, 22, 23), makes the maize–FAW system a suitable candidate for EPN application. Moreover, EPNs exhibit exceptional virulence against FAW with the ability to kill young FAW caterpillars within 24 h (24–27), which is considerably faster than entomopathogenic fungi (10, 28), viruses (11, 29–31), or parasitoids (32). This makes it possible to apply a far lower dosage of EPNs than those normally applied against belowground pests. Furthermore, the isolation of highly virulent EPN strains from local soils eliminates the need for introducing nonnative organisms (27, 33), thereby avoiding potential regulatory barriers.

In a recent laboratory study and pilot field experiment, we demonstrated the potential of EPNs formulated in a cost-effective gel designed to specifically target FAW caterpillars feeding on maize (34). The preliminary field trial with just a single application of the EPN-gel formulation suggested that it can reduce FAW infestation and plant damage as effectively as cypermethrin, a commonly used contact insecticide (35). These results prompted us to conduct the present full-scale study to determine whether EPN treatments can control FAW as effectively as pesticides and to assess their effects on yield. Here, we report on the successful application of the EPN gel throughout an entire maize growing season in Rwanda, encompassing differing climatic conditions and infestation rates. We compare FAW presence and damage as well as maize yield in control plots and plots that were treated with either our in-house developed EPN-gel formulation, a commercial liquid EPN formulation, or cypermethrin. These findings demonstrate that EPNs formulated in a biodegradable gel represent a safe and effective alternative to insecticides for FAW control, particularly in smallholder farms.

## Results

### Plant damage

**Fig. 1.**
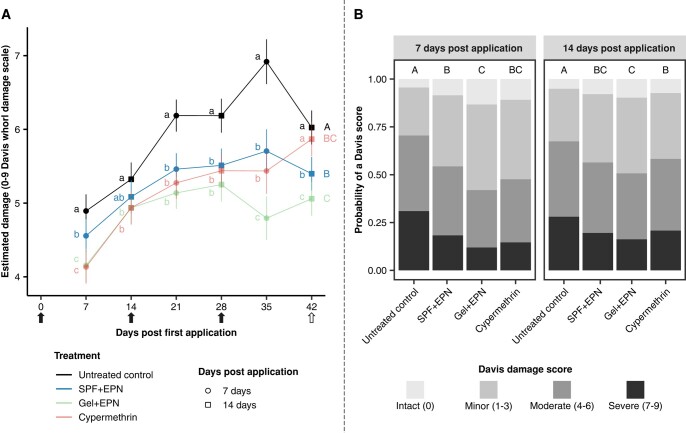
Whorl damage. A) Estimated marginal means ± 95% CI of recorded whorl damage scores averaged for the six fields with each five plots per treatment. The arrows indicate the days of the treatments (black arrows: all fields treated; white arrow: only fields I, II, III, and IV treated). B) Probability of a given damage score within each treatment 7- or 14 days postapplications averaged for six fields and three applications. Whorl damage was assessed 7 and 14 days after each application for 40 plants per plot (*n* = 30 plots per treatment) using the Davis whorl damage scale (36, 37), where a “0” represents an intact whorl and a “9” represents an almost completely or completely destroyed whorl. Data were analyzed using cumulative link mixed models. The letters indicate significant differences (*P* < 0.05) according to multiple comparisons corrected for false discovery using the Benjamini and Hochberg method (38). Panel A: small letters: differences among treatments at a given assessment; capital letters: differences among treatments throughout the field trials.

The overall comparison of the control methods revealed that the whorl damage of maize differed among treatments (Figs. 1 and S1; treatments: $\chi_{\left(3\right)}^{2}$ = 36, *P* < 0.001; triple interaction [Treatments × Application number × Assessments]: $\chi_{\left(6\right)}^{2}$ = 35.85, *P* < 0.001) and increased during the season (Fig. 1A; $\chi_{\left(2\right)}^{2}$ = 1,037, *P* < 0.001). Throughout the season, untreated plants were consistently more damaged than treated plants, regardless of the treatment (Fig. 1A: see capital letters; *P* < 0.001). Plants treated with the gel-formulated EPN were less damaged than plants treated with EPNs formulated in the surfactant polymer formulation (SPF; *P* < 0.01) and untreated plants (*P* < 0.001). The effect of treatment with cypermethrin was intermediate (Fig. 1A). Overall, the probability that a plant suffered severe damage (Davis score > 6) was 14% for the EPN-gel-treated plants, 18% for the cypermethrin-treated plants, and 19% for the EPN-SPF-treated plants. The highest probability of severe damage was observed in untreated control plants (30%). Hence, the EPN-gel treatment reduced heavy damage by more than 50%.

At all six measured time points (covering 6 weeks starting when the plants were about 3 weeks old), we found that the plants treated with the EPN gel were considerably less damaged than untreated plants (Fig. 1A). This was still evident after the third application (>28 days post first application), when plants treated with the EPN gel were less damaged than all other treatments, including cypermethrin (Fig. 1A: see lowercase letters; at 35 days: gel vs. cypermethrin, *P* < 0.01; gel vs. SPF, *P* < 0.001; gel vs. control, *P* < 0.001; at 42 days: gel vs. cypermethrin, *P* < 0.001; gel vs. SPF, *P* = 0.04; gel vs. control, *P* < 0.001).

Damage was more severe 2 weeks after each treatment than after 1 week (Fig. 1B; $\chi_{\left(1\right)}^{2}$ = 119, *P* < 0.001), suggesting that FAW reinfested the plants after each treatment and that a more frequent application of the treatments should provide even better protection against FAW.

### FAW infestation

**Fig. 2.**
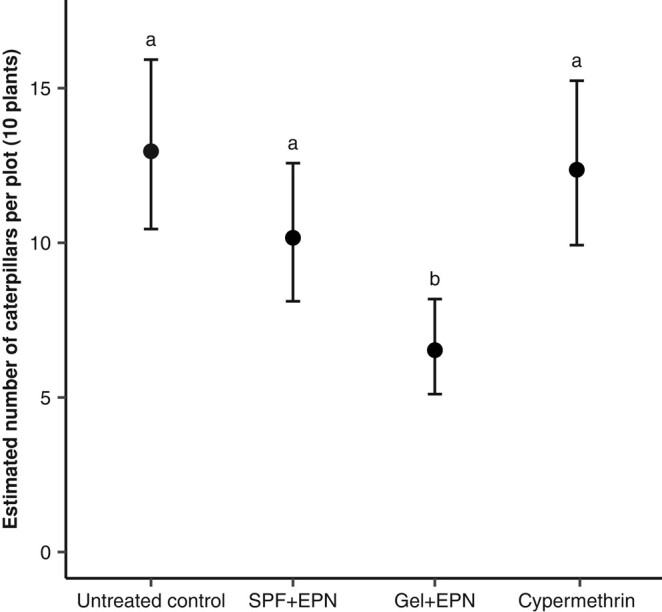
Number of caterpillars per plot. Estimated marginal means ± 95% CI of the number of caterpillars recovered per plot 5 days after the third application, averaged for the six fields with each five plots per treatment. Five days after the third application of the treatments, 10 specific plants per plot (*n* = 30 plots per treatment) were destroyed to inspect for caterpillars. The number of caterpillars (>0.5 cm) was recorded as a proxy for treatment efficacy. Data were analyzed with a generalized linear mixed model using Template Model Builder and a Poisson error distribution. The letters indicate significant differences (*P* < 0.05) according to a multiple comparison corrected for false discovery using the Benjamini and Hochberg method (38).

After the third application of the treatments, we dissected a cohort of 10 plants in the center of each plot to evaluate FAW infestation levels. Overall, the number of caterpillars recovered differed among treatments (Figs. 2 and S2; $\chi_{\left(3\right)}^{2}$ = 22.5, *P* < 0.001), but only plots treated with the EPN formulated in gel were less infested than untreated control plots (gel vs. control: *P* < 0.001). More specifically, on average, 13 ± 1.4 SE caterpillars were found in untreated control plots, 12 ± 1.3 in cypermethrin-treated plots, 10 ± 1.1 in EPN-SPF-treated plots, and 6.5 ± 0.8 in EPN-gel-treated plots. Reinfestation by FAW was prevalent and not affected by any of the treatments, as indicated by the similar occurrence of early instar caterpillars (shorter than 0.5 cm) on plants in all plots (Fig. S3; $\chi_{\left(3\right)}^{2}$ = 5.2, *P* = 0.16).

### Maize yield

**Fig. 3.**
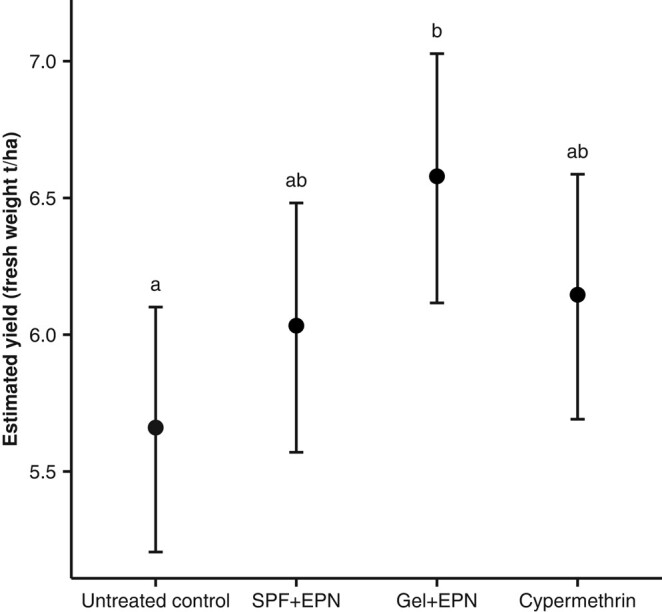
Maize yield. Estimated marginal means ± 95% CI of fresh cob weight averaged from six fields with each five plots per treatment. At harvest, developed cobs from 30 plants in the center of each plot (*n* = 30 plots per treatment) were weighed. Data were analyzed with a linear model and a Gaussian error distribution. The letters indicate significant differences (*P* < 0.05) according to a multiple comparison corrected for false discovery using the Benjamini and Hochberg method (38).

There was an overall treatment effect on yield (Figs. 3 and S4; fresh cob weight, $\chi_{\left(3\right)}^{2}$ = 8.3, *P* = 0.04). At the end of the cropping season, plots that were repeatedly treated with the EPN gel produced almost one additional ton of cobs (fresh weight [FW]) per hectare when compared with untreated control plots (6.6 ± 0.2 SE t/ha vs. 5.7 ± 0.2 t/ha; *P* = 0.03). Cypermethrin-treated plots yielded 6.1 ± 0.2 t/ha, and plots treated with the EPN formulated in SPF produced 6.0 ± 0.2 t/ha, neither one being significantly different from the control plots nor from the plots treated with the EPN gel.

The cobs themselves suffered little FAW damage (∼60% intact; ∼35% little damage [<5% damaged kernels]; <4% medium to heavy damage [>5% damaged kernels]), regardless of whether plants had been treated or not (Fig. S5). Cob damage, as well as the number of cobs produced, was similar in all treatments (Figs. S5 and S6; cob damage: $\chi_{\left(3\right)}^{2}$ = 4.5, *P* = 0.21; number of cobs: $\chi_{\left(3\right)}^{2}$ = 4.5, *P* = 0.21; number of fully developed cobs only: $\chi_{\left(3\right)}^{2}$ = 1.7, *P* = 0.65).

## Discussion

We show that EPNs can be a promising alternative to insecticides for a more sustainable control of FAW. Unlike pesticides, EPNs pose no harm to farmers, consumers, or livestock and are safe for the environment (18). Given the complex nature of the infection process by EPNs and their symbiotic bacteria in an infected insect, it is highly unlikely that FAW is able to develop resistance to EPNs (15, 39, 40). In contrast, several populations of FAW have readily developed resistance to a variety of chemical insecticides (41) as well as to *Bacillus thuringiensis* toxins expressed in transgenic maize (42–44). Another advantage of EPNs is that highly virulent strains can be locally isolated and produced around the world (17, 33), avoiding the introduction of nonnative organisms, reducing registration hurdles, and providing the opportunity to use nonpatented native biological control agents without having to rely on external sources.

Importantly, the EPN-gel application is expected to be cost-effective, since we used doses of EPN across the whole season that were at least 3-fold lower than what is commonly used to control belowground pests (0.4–0.6 vs. 2–4 billion EPN per ha (45, 46)). Based on our estimations, the amount of gel-formulated EPN used in this study over the season would cost a farmer ∼54 or 72 USD (representing three or four applications). Considering an average retail sale price of 354 USD per ton of maize grain in Rwanda (47), as well as the additional ton of cobs produced in plots treated with the EPN-gel formulation when compared with control plots, the net benefit of our approach could be of at least 70 USD (we estimated an additional ton of cobs to represent about 0.4 ton of grains).

The current study shows that repeated application of EPNs during the entire vegetative growing season of maize can consistently reduce plant damage. Both the gel formulation and the surfactant formulation were as effective as the insecticide cypermethrin. The three treatments showed a similar efficacy throughout the first 4 weeks of the field trials (Fig. 1A; plants with up to 10 leaves). After the fifth week, however, the EPN applied in the gel provided considerably better protection, indicating that it was the most effective treatment on older plants (>6-week-old plants; >12 leaves). In contrast, the cypermethrin was found to be less effective after the fourth week and failed to prevent plant damage at 6 weeks (Fig. 1A). This lack of efficacy of the insecticide could be due to heavy rains that followed the last two applications (in fields I, II, III, and IV), which may have washed off or diluted the insecticide, whereas the more viscous gel or the commercial liquid EPN formulation containing surfactants may have remained better on the plants. The fact that the insecticide did not work at the end of the season may also have been an issue with dose. We consistently applied each treatment as a 2-mL spot-spray into the whorl of the plants. This volume of insecticide proved effective for younger plants during the first 4 weeks of the experiments, but it may have been less effective on older plants (>6-week-old plants). The application covered the entire whorl area (2–3 youngest leaves) but not the lower leaves, which may have harbored some caterpillars in the case of the older plants. In contrast, the gel treatment, which was applied similarly to the insecticide, consistently prevented damage, and significantly reduced FAW infestation, even on older plants (Figs. 1 and 2). The lower number of caterpillars recovered on gel-treated plants may indicate that EPN migrated to lower leaves of the plants, where they could infest additional caterpillars. Indeed, we observed living EPN inside the shoot of dissected maize plants 2 weeks after their application.

Importantly, the trials were conducted at multiple field sites, characterized by different environmental conditions, landscapes, and pest pressures. Furthermore, the results are in agreement with our preliminary field trials conducted in 2020 (34), strongly suggesting that the EPN-gel formulation can be effective in different agricultural contexts and under various environmental conditions. Still, the range of conditions that have been tested so far is limited, and it needs to be recognized that under different environmental and biotic pressures, the formulation may vary in its efficacy.

Throughout the field trials, the plants were subject to high FAW infestation rates (i.e. more than one caterpillar per plant, with about 75% infested plants at “Hillside” and 100% at “Station” and “Forest” locations, in untreated control plots). Plant damage was more severe 2 weeks after each treatment than after 1 week regardless of the treatment (Fig. 1B). This implies that FAW rapidly reinfested plants after each treatment. Hence, under such high pest pressures, a weekly application of the treatment may provide even better protection. This may be different under other climatic and agronomic conditions. Despite reinfestations, applying the EPN gel every two weeks resulted in a significant increase in yield, when compared with untreated control plots (Fig. 3). The additional ton of maize produced readily compensates for the estimated cost of the EPN and should be a major incentive for African farmers to use this safer alternative instead of chemical insecticides. Apart from a recent study (48), previous attempts in field conditions to control FAW with EPN applied with water or in combination with surfactants have failed, indicating that the gel is key to the success of EPN application against FAW (24, 34, 49, 50). Further effort should focus on fine-tuning the application frequency of EPN in accordance with local maize phenology, pest population dynamics, and environmental conditions. In addition, we believe that the efficacy of EPN could be increased by recent developments with regards to their formulation. For instance, an innovative strategy has been developed to coat individual EPN with nanoparticles of titanium dioxide (51), which provides them with an increased tolerance to UV radiation (52).

We envision that local biocontrol companies could produce native EPN (17) and incorporate the EPN gel in large caulking gun-like devices specifically designed for smallholder farmers, allowing them to rapidly treat their maize plots with a simple and reusable technology. Another approach would be to train farmers in the mass production of EPN, for example, using black soldier fly larvae (53), so that they could use their own, on-farm produced, biological control agents without relying on external sources. In this context, a simplified and self-sufficient method for farmers to produce EPN in vivo without the need for a laboratory was recently described (54). For large-scale commercial farming, high-precision machinery could be developed that applies low doses of EPN-containing gel on maize plants.

To conclude, the Rwandan field trials demonstrate over a complete maize growing season that the application of EPN can be an effective and safe alternative to synthetic insecticides in the fight against FAW and can increase grain yields. The targeted gel application allowed us to use far lower numbers of EPN than what is normally applied to control belowground pest insects, which should make the approach cost-effective for subsistence farmers. Hence, the application of the gel-formulated EPN alone or in combination with other Integrated Pest Management (IPM) practices holds great promise for sustainable maize production and food security in Africa and beyond.

## Materials and methods

### Nematodes and formulations

We used the Rwandan EPN *Steinernema carpocapsae* (strain RW14-G-R3a-2) for this study (55), as we aim to only use local genotypes, and this particular strain has been shown to be highly virulent against FAW (27). EPNs were reared in vivo on the last instar *Galleria mellonella* L. (Lepidoptera: Pyralidae), as described by White (56). Harvested nematodes were stored in the dark at a cool temperature (12 °C) and used within 2 weeks postharvest.

Four plant treatments were compared in this study: (i) untreated control, (ii) application of EPN in a commercially available liquid SPF (e-nema GmbH, Schwentinental, Germany), (iii) application of EPN in a carboxymethyl cellulose (CMC) gel (34), and (iv) a positive control with the application of the pyrethroid contact insecticide cypermethrin. We had previously shown that the formulations by themselves (SPF and gel without EPN) did not affect FAW survival and that the application of EPN applied in just water was only marginally effective (34). We, therefore, excluded these treatments from the here-presented trials.

The SPF and CMC were dissolved in sterile water to a final concentration of 0.11% (as recommended by the provider) and 3% (w/v), respectively. First, 42 g of CMC or 1.54 g of SPF were added to 1,300 mL of water and let to rest. The next day, solutions were vigorously mixed with a whisk until fully dissolved. Then, 2.1 million EPN concentrated in 100 mL of water were added to the formulations and gently mixed. Using a binocular magnifier, we confirmed that the formulations contained about 1,500 EPN/mL (for an application of ∼3,000 EPN per plant in a 2-mL spot-injection/spray). The cypermethrin (Supra EC, ETG Inputs Ltd, India; 50 g a.i./L) was dissolved in water at 1.0 mL/L (∼0.1 mg a.i. applied per plant in a 2-mL spot-spray). The prepared formulations were kept in cool boxes and used within 4 h.

### Field sites

The experiments were carried out in six fields in Southern Rwanda at three locations. Two fields (fields I and II) were located in an agricultural area at the Rwanda Agriculture and Animal Resources Development Board station in Rubona, in the district of Huye (Location Station; GPS: 2°29′00.6″ S, 29°46′29.8″ E; altitude 1,660 m.a.s.l.). Two fields (fields III and IV) were located close to the edge of a natural forest in the district of Huye (Location Forest; GPS: 2°29′10.0″ S, 29°46′57.3″ E; altitude 1,680 m.a.s.l.). The last two fields (fields V and VI) were located in an agricultural terrace landscape on a hillside in the district of Nyamagabe (location Hillside; GPS: 2°30′46.0″ S, 29°29′58.2″ E; altitude 2,140 m.a.s.l.). Throughout the duration of the experiment, the mean temperature during the day (6 AM to 6 PM) at each location was 24.4 ± 5.4, 24.1 ± 5.6, and 22.1 ± 5.2 °C (mean ± SD) and was 17.6 ± 2.7, 18.2 ± 2.4, and 14.1 ± 1.5 °C (mean ± SD) at night (6 PM to 6 AM), for Station, Forest, and Hillside, respectively. In the same order, the average relative humidity recorded was 76.1 ± 18.1, 73.1 ± 18.1, and 77.2 ± 18.0% at these locations.

Fields at the Station and Forest locations measured ∼16 by 42 m, while the fields at the Hillside location measured 5 by 110 m. The two fields at each location were separated by 5 to 10 m. They were planted on 2022 February 17 or 18. The Rwandan maize hybrid RHMN 1,601 was sown every 30 cm in rows separated by 70 cm, representing about 47,000 plants/ha. Fields were fertilized twice with NPK and urea at a rate of 300 or 100 kg/ha, respectively, once before sowing and a second time 4 weeks after sowing, in accordance with local practices. Fields were left untreated against insects until the beginning of the experimental applications. The experiment was carried out between February and June 2022. We used three locations that were spatially separated (locations Forest and Hillside are about 30 km apart), which comprised different environmental conditions (e.g. altitude, exposition, vegetation).

### Experimental design

Each of the 6 fields was divided into 20 plots comprising 6 rows of 12 to 16 plants (∼72–96 plants per plot). Five plots per fields were attributed to one of the four treatments in a systematic block design (30 plots per treatment in total). The four treatments [(i) untreated; (ii) EPN in SPF; (iii) EPN in CMC gel; and (iv) cypermethrin] were prepared as described above. Treatments were applied as 2 mL spot-injections/sprays into the whorl of each plants using 20 mL plastic syringes for the gel, or hand sprayers for the SPF and cypermethrin. The first application occurred when at least 30% of the plants were showing signs of FAW damage (upper recommended action threshold (57)), and treatments were repeated every 2 weeks until flowering. In fields I, II, III, and IV (“Rubona” and Forest locations), the first application occurred 18 days postsowing (about 90% of plants damaged), when plants carried 4–5 leaves. In total, four applications were performed in these fields (Rubona and Forest locations). In fields V and VI, which were higher in altitude and infested at a later stage, the first applications occurred 38 days postsowing (about 35% of plants damaged), when plants carried 7–9 leaves. In total, three applications were done in the Hillside fields, again until the maize started to flower. All plants within a plot were treated, but only the plants in the four central rows (and at least 1.5 m away from the next plot) were used for data collection.

### Data collection

Seven and 14 days after the first, second, and third applications, plant damage was visually assessed for 40 plants in the center of each plot using the Davis whorl damage scale (36), as described in Toepfer et al. (37). Briefly, the Davis scale is a top-view assessment, where only the damage to the youngest leaves of the whorl is evaluated, where FAW caterpillars are mostly feeding. Hence, the damage recorded is noncumulative, as old damages located on lower leaves are not considered. The Davis whorl damage scale ranges from “0” to “9,” whereby a fully intact whorl is attributed a score of “0,” while an almost totally destroyed whorl is attributed a score of “9.” Minor damages are represented by scores ranging from 1 to 3, while moderate or severe damages by scores ranging from 4 to 6 or 7 to 9, respectively. In total, 40 plants from the four central rows of a plot were assessed at both 7 and 14 days postapplications (200 plants per treatment and field). Plant damage was evaluated by 2 assessors per plot (each evaluating 20 plants in 20 plots per field). Plant damage was not assessed after the fourth application (fields I, II, III, and IV), as plants were too tall to visually inspect the whorl without damaging the plants.

Five days after the third application (second-to-last application in fields I, II, III, and IV; last application in fields V and VI), we also evaluated the presence of FAW caterpillars on the plants. For this, 10 specific plants within the 4 central rows of each plot were systematically destroyed to inspect the plants for caterpillars. The occurrence (yes/no) of young caterpillars (shorter than 0.5 cm) was determined as a proxy for reinfestation, whereas the number of older caterpillars (longer than 0.5 cm) was used as a proxy for treatment efficacy. The dissection of the plants and search for caterpillars were done by several assessors who had no knowledge of the treatment being evaluated, but each assessed plants from all treatments.

At harvest, the number of fully developed and underdeveloped cobs from the 30 remaining plants in the center of each plot was recorded. The fully developed cobs were collected and husked to assess cob damage, which was evaluated visually using a “0” to “3” customized damage score. A score of “0” represents an intact cob, while scores of “1,” “2,” or “3” represent minor (<5% damaged kernels), moderate (5–20% damaged kernels), or severe (>20% damaged kernels) cob damage, respectively. The husked cobs from the 30 plants were then weighed together to estimate FW yield per plot and evaluate treatment effects on yield. For each plot, the assessments were carried out by 2 assessors who had no knowledge of the treatment being evaluated.

### Data analyses

Statistical analyses were performed using R version 4.2.1 (58). For each analysis, fields were pooled in a single dataset and included in the statistical models as fixed factors to account for the local variation among field sites. The results presented are estimated marginal means from the models that show the average effect of the treatments across the different sites.

Plant damage was analyzed using cumulative link mixed models (“ordinal” package (59)), followed by multiple comparisons (“emmeans” package (60)) corrected for false discovery using the Benjamini and Hochberg (38) method. The damage score given to a plant was used as the response variable. Treatments, the application number (4 applications), as well as assessment times (7 or 14 days posttreatment) were used as fixed factors, plus their triple interactions. Field number (six fields) and assessors were used as fixed factors, while plots and plants were used as random factors. The damage score probabilities reported were calculated using the function “rating.emmeans” (“RVAideMemoire” package (61)).

Cob damage was analyzed using cumulative link mixed models (“ordinal” package (59)), similarly as plant damage. The damage score given to a cob was used as the response variable, while treatments and field number (six fields) were used as fixed factors. Plots were used as a random factor.

Maize yield was analyzed using a linear model with a Gaussian error distribution. Yield per plot was used as the response variable, while treatments and field numbers (six fields) were used as fixed factors. The number of caterpillars (longer than 0.5 cm) per plot, the total number of all cobs (underdeveloped + developed), as well as the number of developed cobs only were analyzed similarly but using generalized mixed models with a Poisson error distribution. Plots were included here as a random factor to correct for overdispersion. The occurrence of young caterpillars (shorter than 0.5 cm) per plant was analyzed similarly, with a binomial error distribution. Plots were used as a random factor to account for the repeated observations within a plot. The function “glmmTMB” (“glmmTMB” package (62)) was used to perform the models, while the function “Effect” (“effects” package (63)) was used to compute the model estimates presented in the figures and the Results section. A multiple comparison (“emmeans” package (60)) corrected for false discovery using the Benjamini and Hochberg (38) method was performed to compare treatments when they had a significant effect.

## Supplementary Material

pgae122_Supplementary_Data

## Data Availability

The source data and code supporting the findings of this study are available on Zenodo at: https://doi.org/10.5281/zenodo.10117988.
