# Supplementary material for: Entomopathogenic nematodes as an effective and sustainable alternative to control the fall armyworm in Africa
Source: PNAS Nexus. 2024 Apr 16;3(4):pgae122. doi: 10.1093/pnasnexus/pgae122 (PMC11020222; doi:10.1093/pnasnexus/pgae122)
Supplement: pgae122_Supplementary_Data [file pgae122_supplementary_data.docx]

**
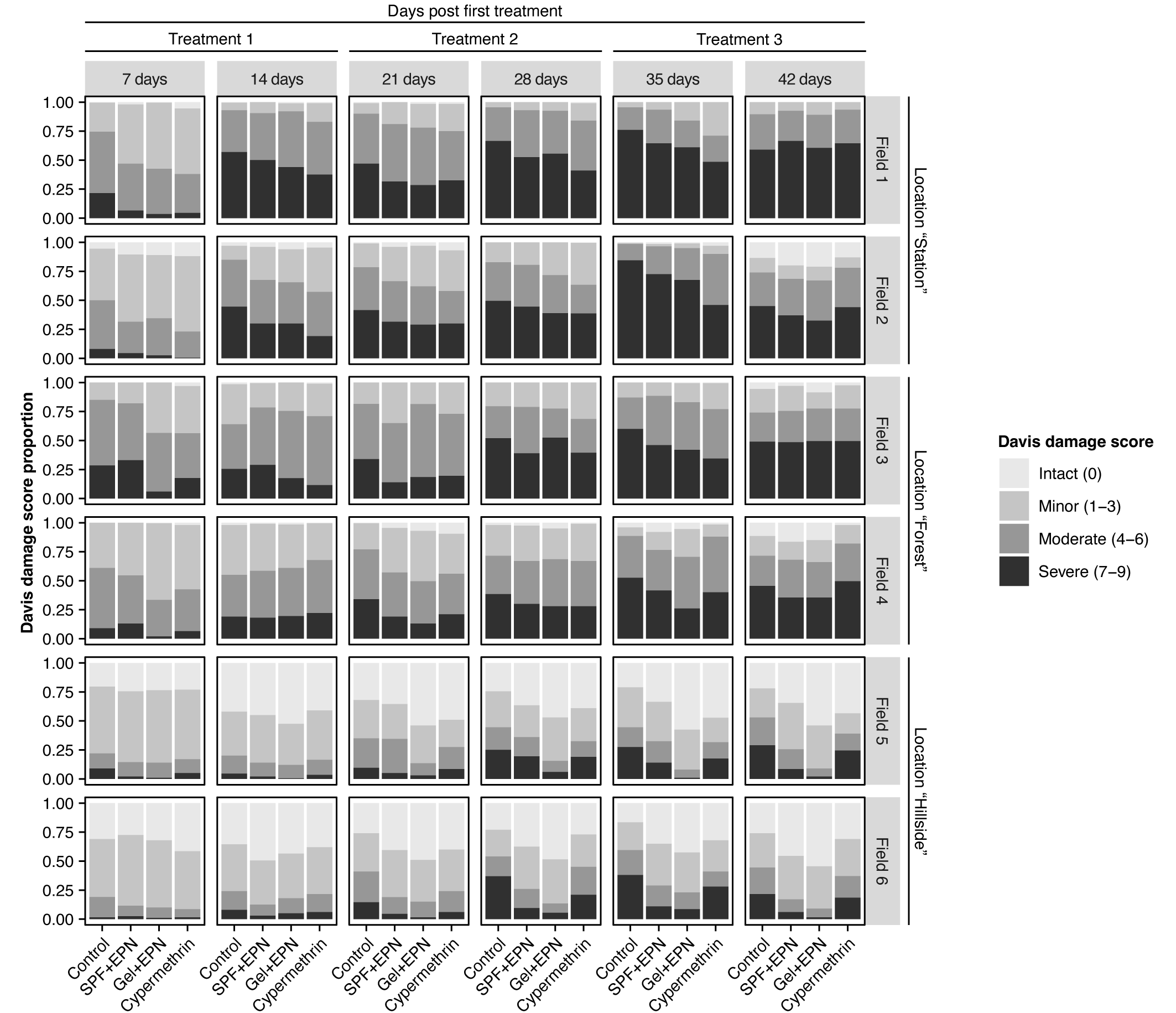
**

**Figure S1. Recorded whorl damage at six assessments and in six fields.** Whorl damage was assessed seven- and fourteen-days after each application for 40 plants per plot (n = 200 plants per treatment, assessment and field) using the Davis whorl damage scale, where a “0” represents an intact whorl and a “9” represents an almost completely or completely destroyed whorl.


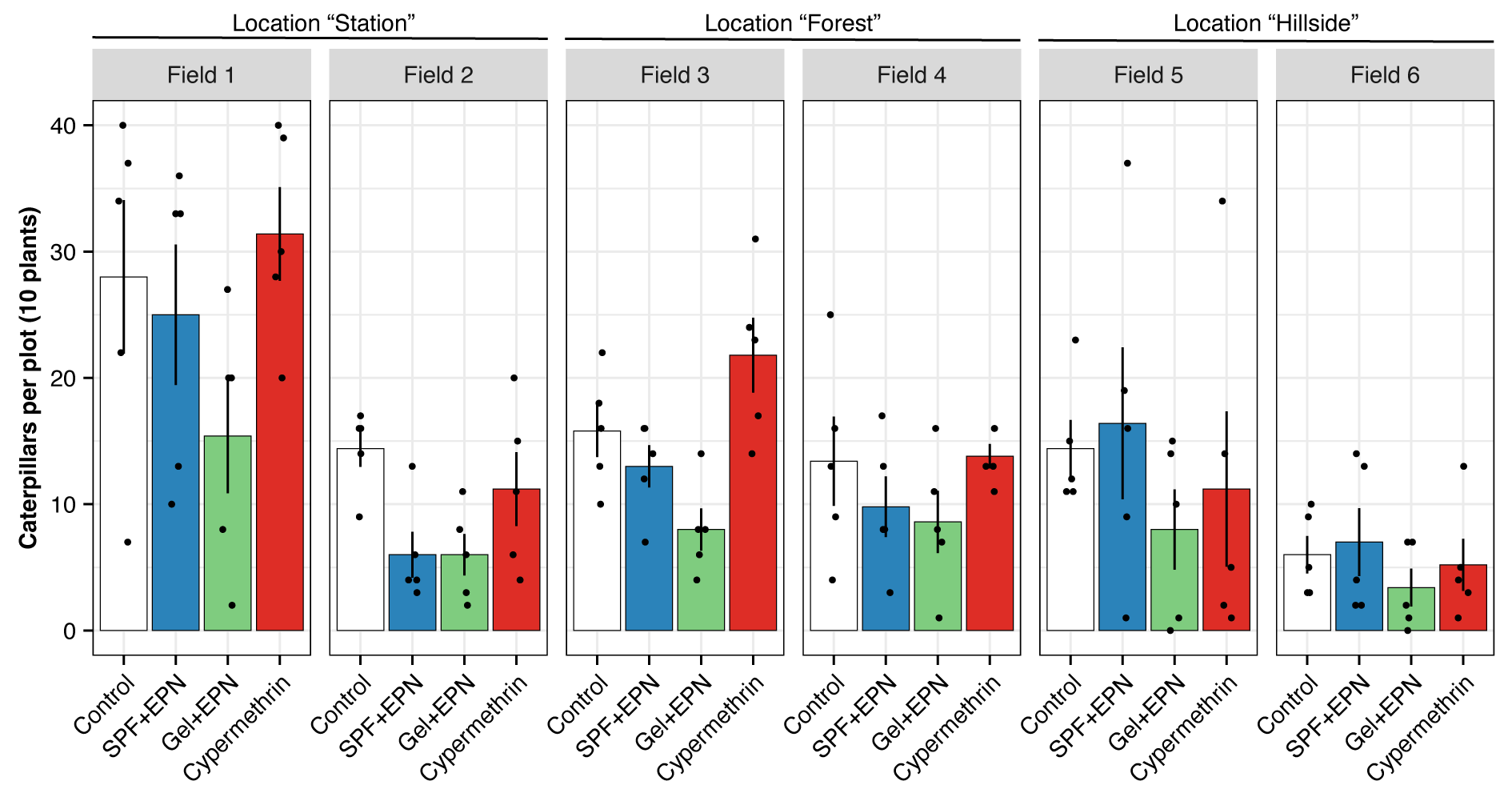


**Figure S2. Average number of caterpillars (± SE) per treatment and field.** Five-days after the third application of the treatments, ten specific plants per plot (n = 5 plots per treatment and field) were destroyed to inspect for caterpillars. The number of caterpillars (bigger than 0.5 cm) was recorded as a proxy for treatment efficacy. Dots indicate the number of caterpillars recovered per plot.


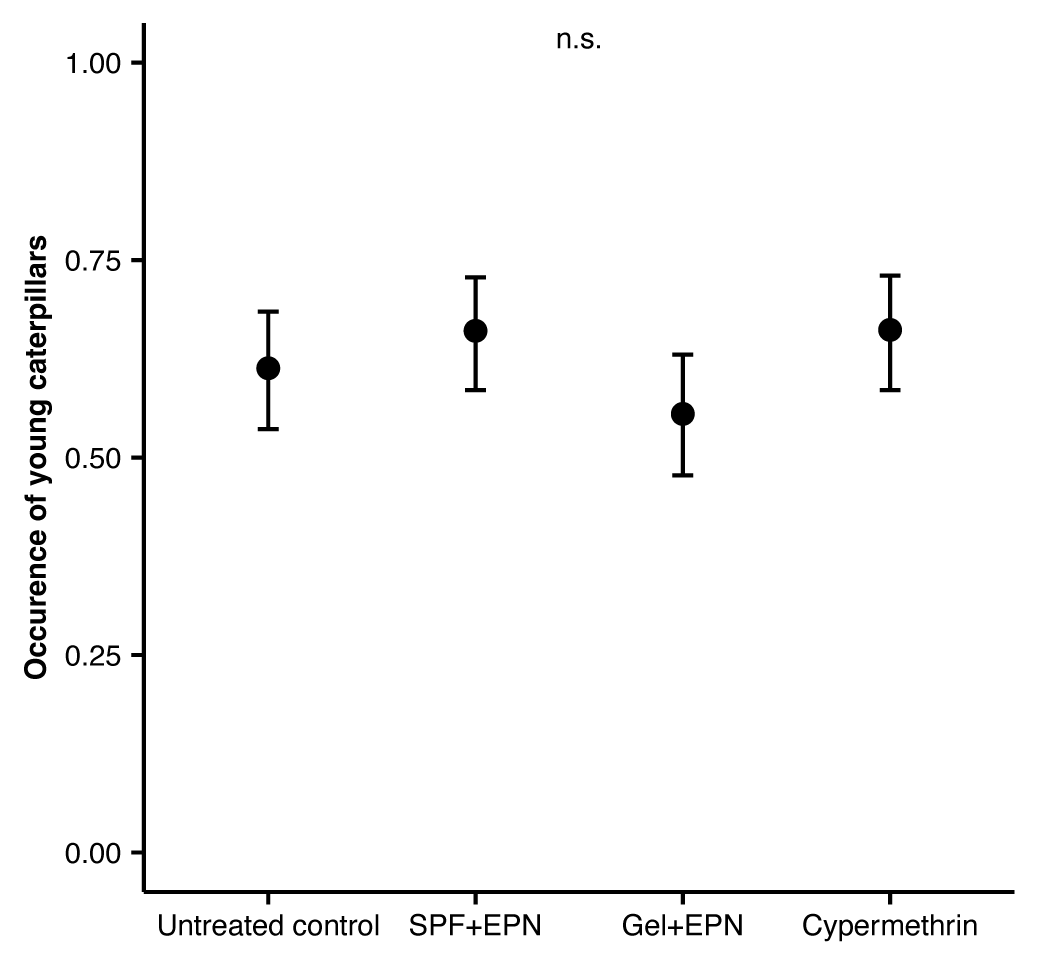


**Figure S3. Occurrence of young caterpillars on plants, indicating rapid re-infestation by the fall armyworm.** Estimated marginal means ± 95% CI of the occurrence of young caterpillars (smaller than 0.5 cm) recorded per plant and averaged from five plots in each of six fields. Five-days after the third application of the treatments, ten specific plants per plot (n = 30 plots per treatment) were destroyed to search for caterpillars. The occurrence of young caterpillars (smaller than 0.5 cm) was recorded as a proxy for reinfestation. Data were analysed with a generalized linear mixed model using Template Model Builder and a binomial error distribution. “n.s.” indicates non-significant differences (p > 0.05).


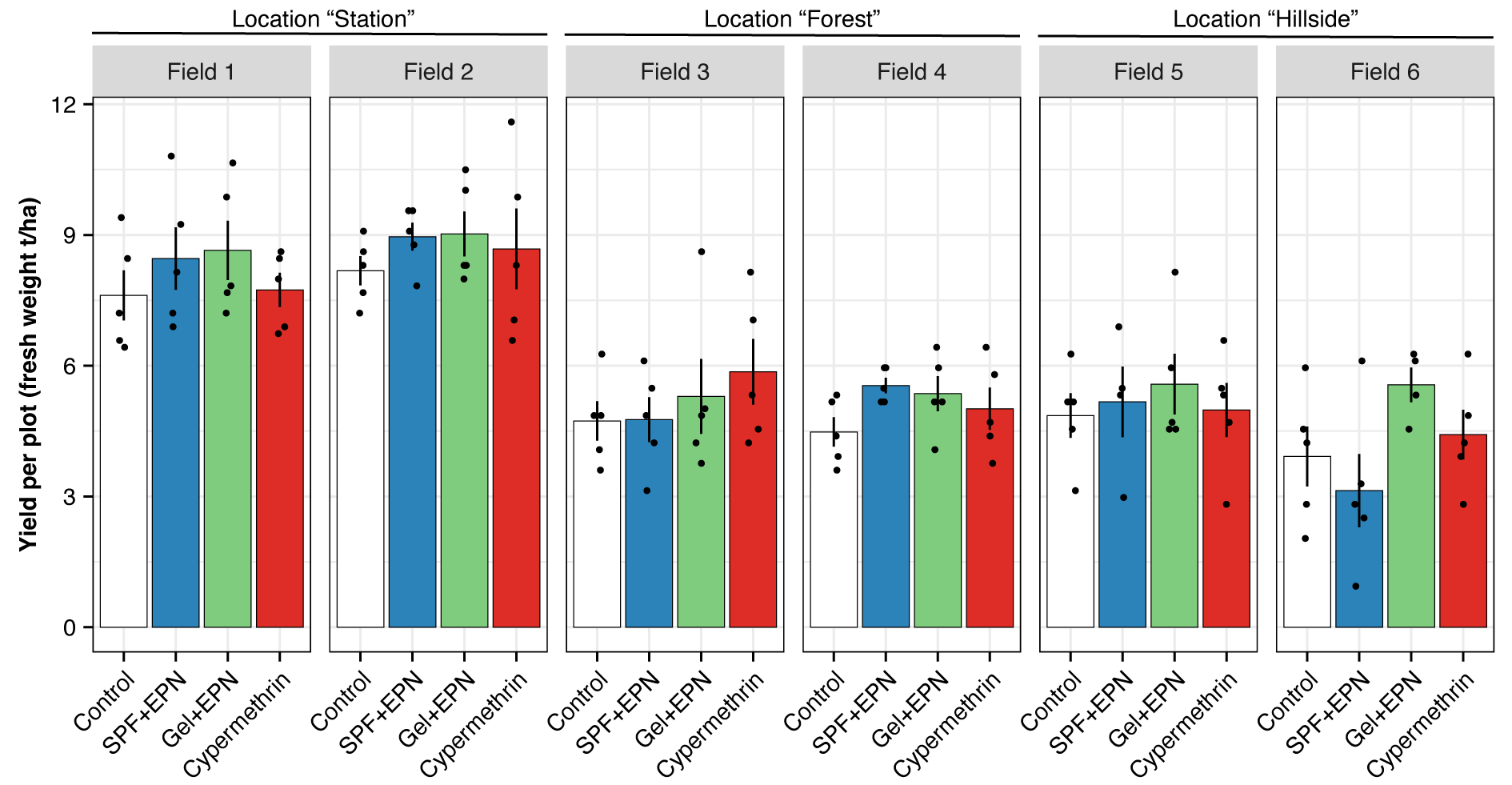


**Figure S4.** **Average fresh weight cob yield (± SE) per treatment and field.** At harvest, developed cobs from 30 plants in the centre of each plot (n = 5 plots per treatment and field) were weighed. Dots indicate yield per plot.


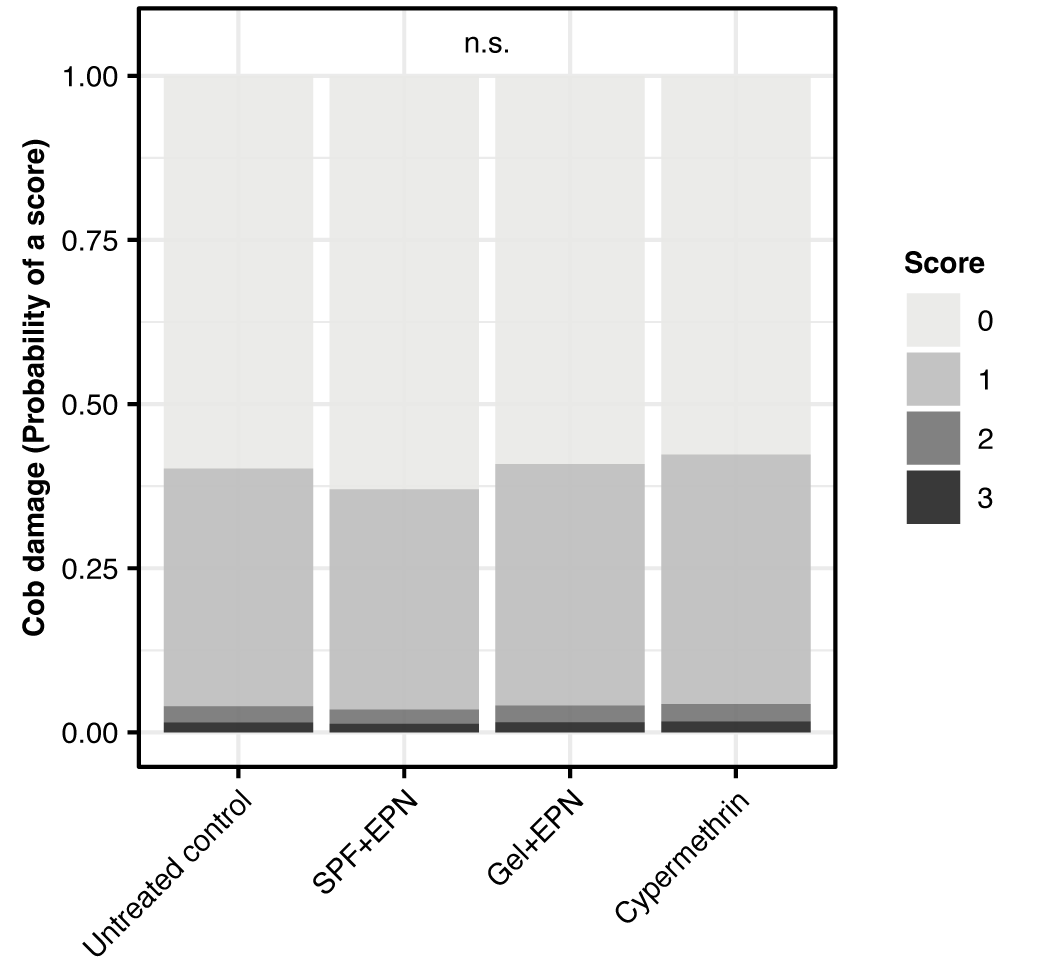


**Figure S5. Cob damage.** Probability of a given damage score within each treatment averaged for six fields, with each five plots per treatment. Cob damage was assessed on developed cobs from 30 plants in the centre of each plot (n = 30 plots). A score of “0” represent an intact cob, while scores of “1”, “2”, or “3” represent minor (<5% damaged kernels), moderate (5-20% damaged kernels) or severe (>20% damaged kernels) cob damage, respectively. Data were analysed using a cumulative link mixed model. “n.s.” indicates non-significant differences (p > 0.05).


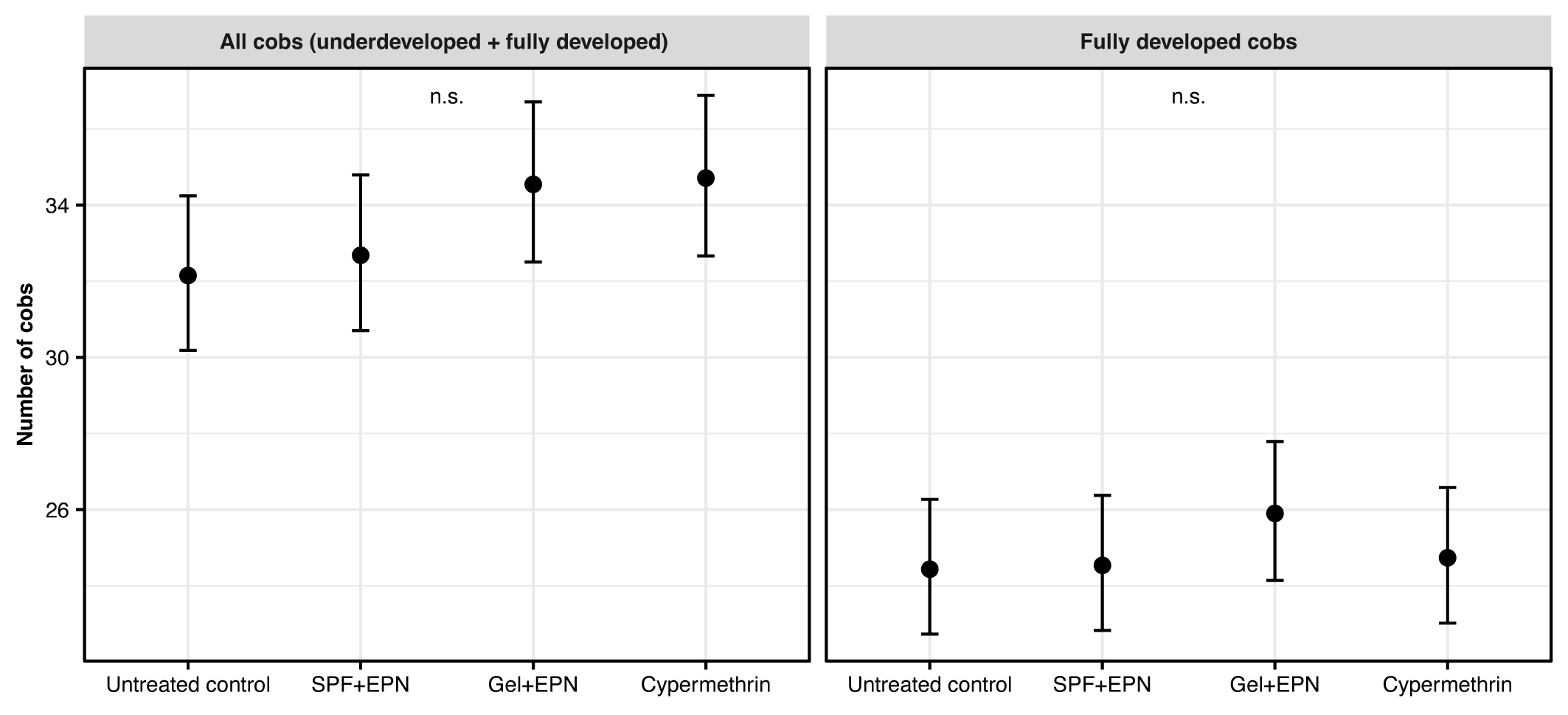


**Figure S6. Number of cobs per plot.** Estimated marginal means ± 95% CI of the number of cobs recorded per plot and averaged from six fields, with each five plots per treatment. At harvest, underdeveloped as well as developed cobs from thirty plants in the centre of each plot (n = 30 plots per treatment) were counted. Data were analysed with generalized linear mixed model using Template Model Builder and poisson error distribution. “n.s.” indicates non-significant differences (p > 0.05).
